# Association of *BET1L* and *TNRC6B* with uterine leiomyoma risk and its relevant clinical features in Han Chinese population

**DOI:** 10.1038/s41598-018-25792-z

**Published:** 2018-05-09

**Authors:** Bailing Liu, Tao Wang, Jue Jiang, Miao Li, Wenqi Ma, Haibin Wu, Qi Zhou

**Affiliations:** 1 Department of Ultrasound, the Second Affiliated Hospital, Xi’an Jiaotong University, Xi’an, Shaanxi China; 2 Department of Ultrasound, Children’s Hospital of Xi’an, Xi’an, Shaanxi China; 3 Department of Pediatrics, Children’s Hospital of Xi’an, Xi’an, Shaanxi China

## Abstract

Previous studies have shown that uterine leiomyomas (UL) are benign tumours with contributions from environmental and genetic factors. We aimed to replicate two initial significant genetic factors, *TNRC6B* and *BET1L*, in a Han Chinese population. A total of 2,055 study subjects were recruited, and 55 SNPs mapped to *TNRC6B* and *BET1L* were selected and genotyped in samples from these subjects. Genetic associations were analysed at both the single marker and haplotype levels. Associations between targeted SNPs and relevant clinical features of UL were analysed in case only samples. Functional consequences of significant SNPs were analysed by bioinformatics tools. Two SNPs, rs2280543 from *BET1L* (χ^2^ = 18.3, OR = 0.64, *P* = 1.87 × 10^−5^) and rs12484776 from *TNRC6B* (χ^2^ = 19.7, OR = 1.40, *P* = 8.91 × 10^−6^), were identified as significantly associated with the disease status of UL. Rs2280543 was significantly associated with the number of fibroid nodes (*P* = 0.0007), while rs12484776 was significantly associated with node size (χ^2^ = 54.88, *P* = 3.44 × 10^−11^). Both SNPs were a significant eQTL for their genes. In this study, we have shown that both *BET1L* and *TNRC6B* contributed to the risk of UL in Chinese women. Significant SNPs from *BET1L* and *TNRC6B* were also identified as significantly associated with the number of fibroid nodes and the size of the node, respectively.

## Introduction

Uterine leiomyomas (UL), also known as uterine fibroids, are benign tumours in the uterus^1^. UL are typically identified during the mid or late reproductive years in women, and they decrease in size after menopause^2^. The size and number of fibroids can vary among patients^3^. According to a systematic review published in 2017, the incidence of UL could be ranged from 217–3745 cases per 100,000 women-years^4^. In general, the prevalence of UL in women ranges from 20% in Europeans to as high as 80% in African-American women^4,5^. No symptoms can be identified in more than 50% of women with UL^6^. For the remaining women with UL, their clinical symptoms can range from abnormal bleeding and pelvic pain to infertility and pregnancy complications^6^.

Evidence from multiple studies has shown that UL have contributions from both environmental and genetic factors^7–9^. Early familial aggregation and twin studies have identified a significant genetic component to UL predisposition^10,11^. Makinen *et al*. performed a whole-exome sequencing study on 18 UL patients and identified the *MED12* gene as contributing to tumourigenesis^12^. In a GWAS based on Japanese populations conducted in 2011, three loci on chromosomes 10q24.33, 22q13.1, and 11p15.5 were identified to be significantly associated with the disease status of UL^13^. These three loci included several genes such as STE20 Like Kinase (*SLK*), Oligosaccharide-Binding Fold-Containing Protein 1 (OBFC1), Trinucleotide Repeat Containing 6B (*TNRC6B*), Outer Dense Fiber 3 (*ODF3*), Bet1 Golgi Vesicular Membrane Trafficking Protein Like (*BET1L*), RIC8 Guanine Nucleotide Exchange Factor A (*RIC8A*), and Sirtuin 3 (*SIRT3*). Since then, several follow up studies have tried to replicate these initial GWAS findings using study samples based on other ethnic groups^14–16^. However, the results of these subsequent studies have not been concordant and have, at times, been contradictory. More studies with large sample sizes are still needed to confirm these previous hits.

In this study, we attempted to replicate two initial significant loci, *TNRC6B* and *BET1L*, identified in a GWAS conducted by Cha *et al*.^13^ by using study subjects with Chinese Han ancestry. A total of 2,055 study subjects were recruited, and 55 SNPs mapped to *TNRC6B* and *BET1L* were selected and genotyped in samples from these subjects. In addition to genetic associations between these SNPs and the disease status of UL, we also examined potential associations between targeted SNPs and clinical characteristics of UL. Bioinformatics tools were also utilized to evaluate the potential biological functions of the targeted SNPs.

## Methods

### Study subjects

In the present study, a total of 674 women with UL and 1,381 healthy women, controls without any systematic disease, were recruited from the Second Affiliated Hospital of Xi’an Jiaotong University between April 2013 and May 2017. All patients were diagnosed with UL by ultrasonography and confirmed by at least two senior physicians, and all subjects were screened for no other female reproductive system tumours, systemic disease or history of malignancy. Self-administered questionnaires were used to collect demographic data, and the characteristics of our study subjects are shown in Table 1. All participants were unrelated Han Chinese individuals, and the UL and control groups were matched by age and body mass index (BMI). Significant differences were identified for duration of menses (*P* = 0.005) and menstrual cycle (*P* = 0.003) between UL cases and healthy controls. The size of UL was categorized into three groups (small, medium, and large) based on the diameter of the UL (small ≤2 cm, 2 cm <medium <4 cm, large ≥4 cm). If subjects were diagnosed with multiple UL, the largest one determined the size group. The study protocol was approved by the Ethics Committee of Xi’an Jiaotong University in accordance with the ethical guidelines of the Declaration of Helsinki of 1975 (revised in 2008). Written informed consent was obtained from participants.

**Table 1:** The clinical and demographic characteristics of the uterine leiomyoma and control groups.

Characteristics	Subjects (N = 2,055)		*P*-value
	UL patients (N = 674)	Controls(N = 1,381)	
Age, mean ± SD (years)	38.3 ± 7.22	38.2 ± 7.34	0.78
BMI, mean ± SD (kg/m^2^)	23.7 ± 1.37	23.6 ± 1.30	0.12
Married (%)	647 (96.0)	1328 (96.2)	0.94
Age of menarche, mean ± SD (years)	13.5 ± 1.47	13.4 ± 1.38	0.12
Duration of menses, mean ± SD (days)	6.5 ± 1.52	6.7 ± 1.69	0.005
Menstrual cycle, mean ± SD (days)	28.8 ± 1.39	28.6 ± 1.67	0.003
Bleeding (%)	396 (58.5)	NA	NA
Pain (%)	225 (33.4)	NA	NA
Fibroid node (%)		NA	NA
Single	228(33.8)		
Multiple	446(66.2)		
Node size (%)		NA	NA
Small	79(11.7)		
Medium	434(64.4)		
Large	161(23.9)		

UL: uterine leiomyoma; BMI: body mass index; SD: standard deviation; NA: not available.

### SNP selection and Genotyping

We searched for all SNPs with a minor allele frequency (MAF) ≥0.05 within the regions of the *TNRC6B* and *BET1L* genes in the 1000 Genomes Chinese Han Beijing population (CHB). Then, MAF ≥0.05 with pair-wise tagging and r^2^ ≥0.8 were used as the cut-off criteria during tag SNP selection, which generated 27 and 28 tag SNPs within the *TNRC6B* and *BET1L* genes, respectively. General information about these 55 selected SNPs is summarized in Supplemental Table S1. Most of the selected SNPs were non-coding SNPs. Genomic DNA was extracted from peripheral blood leukocytes according to the manufacturer’s protocol (Genomic DNA kit, Axygen Scientific Inc., California, USA). Genotyping was performed for all SNPs using the Sequenom Mass ARRAY RS1000 system (Sequenom, San Diego, California, USA). The results were processed using Typer Analyser software, and genotype data were generated from the samples^17^. Case and control status was blinded during all genotyping processes for quality control. Five percent of the samples were repeated at random, and the results were 100% concordant.

### Statistical and Bioinformatics Methods

Hardy-Weinberg equilibrium was tested for each SNP within the control samples. χ^2^ tests were performed for each SNP to evaluate the differences in allelic and genotypic distributions between UL cases and controls. Linkage disequilibrium (LD) blocks were constructed for both genes, and haplotype-based analyses were conducted for each block. Plink was utilized for the analyses mentioned above^18^. In addition to genetic association analyses focusing on disease status, we also analysed the potential link between significant SNPs and four clinical features of UL, including bleeding, pain, number of fibroid nodes, and size of the node, in a subset of our samples that included UL cases only. χ^2^ tests were performed for these analyses. In general, Bonferroni correction was applied to address multiple comparisons. For single marker-based association analyses, the threshold *P* value was 0.05/55 ≈ 9 × 10^−4^. Genomic control was applied to correct for the potential effects of population stratification^19,20^. The null distribution of genomic inflation factor λ was constructed by 10,000 bootstrapping.

The potential biological functions of our selected SNPs were evaluated through RegulomeDB (http://www.regulomedb.org/)^21^. RegulomeDB is a database that annotates SNPs based on known and predicted regulatory element data from the ENCODE project. A score ranging from 1–6 was assigned to each SNP, and a lower score indicated a more significant biological function. In addition, we also extracted eQTL data from the GTEx database (https://www.gtexportal.org/home/)^22^ to examine differences in gene expression associated with our significant SNPs.

## Results

We identified two significant SNPs in our two candidate genes: rs2280543 in *BET1L* (3′-untranslated region, χ^2^ = 18.3, OR = 0.64, *P* = 1.87 × 10^−5^) and rs12484776 in *TNRC6B* (Intron, χ^2^ = 19.7, OR = 1.40, *P* = 8.91 × 10^−6^) (Table 2 and Supplemental Table S2). Genotypic analyses verified this result. Genomic controls applied on the results of single marker-based association analyses showed no significant inflations in χ^2^ statistics. The inflation factor was less than 1, as was the upper boundary of the 95% confidence interval (Supplemental Figure S1). Six LD blocks were constructed for *BET1L*, and another seven blocks were constructed for *TNRC6B*. Haplotype-based analyses identified 2 significant two-SNP LD blocks (Table 3). LD block rs2280543-rs4980319 in *BET1L* (χ^2^ = 77.56, *P* = 1.44 × 10^−17^) and LD block rs12485003-rs12484776 in *TNRC6B* (χ^2^ = 27.69, *P* = 9.70 × 10^−7^) were identified to be significantly associated with the disease status of UL. Further analyses using UL case only samples identified rs2280543 as significantly associated with the number of fibroid nodes (*P* = 0.0007), while rs12484776 was significantly associated with the size of the node (χ^2^ = 54.88, *P* = 3.44 × 10^−11^) (Table 4).

**Table 2:** Genetic association of rs2280543 and rs12484776 with UL.

		Allelic Test					Genotypic Test				
rs2280543(%)		T (N = 538)	C (N = 3,572)	OR	χ^2^	P*	TT (N = 37)	CT (N = 464)	CC (N = 1,554)	χ^2^**	P*
	Patients	133 (9.8)	1,215 (90.2)				3 (0.4)	127 (18.8)	544 (80.8)		
	Controls	405 (14.6)	2,357 (85.4)	0.64	18.3	1.87 × 10^−5^	34 (2.5)	337 (24.4)	1,010 (73.1)	—	1.85 × 10^−5^
rs12484776(%)		G (N = 999)	A (N = 3,111)				GG (N = 125)	GA (N = 749)	AA (N = 1,181)		
	Patients	385 (28.6)	963 (71.4)				61 (9.1)	263 (39.0)	350 (51.9)		
	Controls	614 (22.2)	2,148 (77.8)	1.40	19.7	8.91 × 10^−6^	64 (4.6)	486 (35.2)	831 (60.2)	21.7	1.94 × 10^−5^

^*^P value threshold after Bonferroni corrections was 0.05/55 ≈ 9 × 10^−4^.

**Fisher exact test was applied due to sparse cell.

**Table 3:** Haplotype based genetic associations of *BET1L* and *TNRC6B* with UL.

LOCUS	SNPS	χ^2^	DF	*P*
*BET1L*	rs201966829|rs13377507	1.42	2	0.4918
*BET1L*	rs11502187|rs118152462	0.71	2	0.7002
*BET1L*	rs3825076|rs3802984|rs3741411	2.16	3	0.5401
*BET1L*	rs75155656|rs11245992	1.24	2	0.5379
***BET1L***	**rs2280543|rs4980319**	**77.56**	**2**	**1.44 × 10** ^**−17**^
*BET1L*	rs3782120|rs7930823	1.35	2	0.5103
*TNRC6B*	rs12628757|rs6001794	0.27	2	0.8739
*TNRC6B*	rs117941537|rs11089974	1.89	2	0.3888
*TNRC6B*	rs739182|rs77943556	8.06	2	0.0178
***TNRC6B***	**rs12485003|rs12484776**	**27.69**	**2**	**9.70 × 10** ^**−7**^
*TNRC6B*	rs57960171|rs743897	0.69	2	0.7085
*TNRC6B*	rs139914|rs4821942	1.35	2	0.5093

DF, degree of freedom. Significant results were highlighted in bold. *P* value threshold after Bonferroni corrections was 0.05/12 ≈ 0.004.

**Table 4:** Genetic associations of rs2280543 and rs12484776 with clinical characteristics of UL patients.

Genotype	rs2280543			χ^2^*	P**	rs12484776			χ^2^	P**
	TT (N = 3)	CT (N = 127)	CC (N = 544)			GG (N = 61)	GA (N = 263)	AA (N = 350)		
Bleeding(%)										
Yes	2 (0.5)	82 (20.7)	312 (78.8)			28 (7.1)	163 (41.2)	205 (51.7)		
No	1 (0.3)	45 (16.2)	232 (83.5)	—	0.2934	33 (11.9)	100 (36.0)	145 (52.1)	5.29	0.071
Pain(%)										
Yes	2 (0.9)	44 (19.6)	179 (79.5)			17 (7.6)	91 (40.4)	117 (52.0)		
No	1 (0.2)	83 (18.5)	365 (81.3)	—	0.4173	44 (9.8)	172 (38.3)	233 (51.9)	1	0.6036
Fibroid node(%)										
Single	0 (0)	27 (11.8)	201 (88.2)			21 (9.2)	101 (44.3)	106 (46.5)		
Multiple	3 (0.7)	100 (22.4)	343 (76.9)	—	**0.0007**	40 (9.0)	162 (36.3)	244 (54.7)	4.43	0.1091
Node size(%)										
Small	0 (0)	14 (17.7)	65 (82.3)			16 (20.3)	9 (11.4)	54 (68.5)		
Medium	1 (0.2)	84 (19.4)	349 (80.4)			19 (4.4)	195 (44.9)	220 (50.7)		
Large	2 (1.2)	29 (18.0)	130 (80.8)	—	0.5808	26 (16.1)	59 (36.6)	76 (47.3)	**54.88**	**3.44 × 10** ^**−11**^

*Fisher exact tests were applied when there were sparse cells and therefore no χ^2^ statistics were reported. Significant results were highlighted in bold.

**P value threshold after Bonferroni corrections was 0.05/8 ≈ 0.00625.

Data extracted from RegulomeDB showed that both SNPs, rs2280543 and rs12484776, had a RegulomeDB score of 5 (Supplemental Table S1). This score indicates that there was very limited evidence indicating the potential regulatory role of these two SNPs. Expression quantitative trait loci (eQTL) data from GTEx for both rs2280543 and rs12484776 were extracted and examined. Significant findings are summarized in Table 5. The threshold P values were 0.05/47 ≈ 0.001. SNP rs2280543 was found to be significantly associated with *BET1L* gene expression in 15 of 47 human tissues, while rs12484776 was identified to be significant only in oesophagus muscularis (effect size = −0.17, *P* = 4.60 × 10^−4^). Neither SNP was significantly associated with gene expression in the uterus (Supplemental Table S3).

**Table 5:** Significant eQTL results for rs2280543 and rs12484776.

GENE	SNP	*P*	Effect Size	T-Statistic	Standard Error	Tissue
*BET1L*	rs2280543	1.70 × 10^−18^	0.73	9.20	0.08	Muscle - Skeletal
*BET1L*	rs2280543	1.10 × 10^−11^	0.53	7.10	0.08	Artery - Tibial
*BET1L*	rs2280543	5.10 × 10^−11^	0.47	6.80	0.07	Skin - Sun Exposed (Lower leg)
*BET1L*	rs2280543	5.40 × 10^−10^	0.58	6.40	0.09	Esophagus - Mucosa
*BET1L*	rs2280543	1.20 × 10^−9^	0.50	6.30	0.08	Esophagus - Muscularis
*BET1L*	rs2280543	5.20 × 10^−8^	0.58	5.60	0.10	Artery - Aorta
*BET1L*	rs2280543	1.40 × 10^−7^	0.45	5.40	0.08	Adipose - Subcutaneous
*BET1L*	rs2280543	1.10 × 10^−6^	0.43	5.00	0.09	Nerve - Tibial
*BET1L*	rs2280543	1.40 × 10^−6^	0.57	5.00	0.12	Breast - Mammary Tissue
*BET1L*	rs2280543	9.50 × 10^−6^	0.91	4.80	0.19	Brain - Spinal cord (cervical c-1)
*BET1L*	rs2280543	1.60 × 10^−5^	0.38	4.40	0.09	Skin - Not Sun Exposed (Suprapubic)
*BET1L*	rs2280543	1.20 × 10^−4^	0.33	3.90	0.09	Thyroid
*BET1L*	rs2280543	2.80 × 10^−4^	0.43	3.70	0.12	Esophagus - Gastroesophageal Junction
*BET1L*	rs2280543	4.00 × 10^−4^	0.46	3.60	0.13	Colon - Sigmoid
*BET1L*	rs2280543	6.80 × 10^−4^	0.57	3.50	0.16	Adrenal Gland
*TNRC6B*	rs12484776	4.60 × 10^−4^	−0.17	−3.50	0.05	Esophagus - Muscularis

*P* value threshold after Bonferroni corrections was 0.05/90 ≈ 5 × 10^−4^.

## Discussion

With the widespread application of sequencing and genetic association analyses for studying the genetics of complex diseases, candidate gene-based association studies have successfully mapped susceptibility for many complex diseases^23–29^. Our data based on ~2000 study subjects from a Chinese Han population provide strong evidence for the genetic association between UL and two candidate genes, *BET1L* and *TNRC6B*. To the best of our knowledge, this study is the first genetic association study for *BET1L* and *TNRC6B* and UL based on Chinese populations. Our findings of single marker-based associations for both rs2280543 and rs12484776 replicate initial reports from Cha *et al*.^13^. Given that it is not sufficient to draw conclusions from limited SNPs analyses^30–32^, we performed haplotype analyses, which indicated a similar pattern with single marker-based associations. However, Bondagji *et al*. performed a replication study based on Saudi women, and rs2280543 from *BET1L* was not reported to be significant^16^. This difference might be due to the different LD structures from different genetic backgrounds. Both Japanese and Chinese Han populations belong to the Asian population and are therefore more genetically similar than Saudi women from the Middle East. In addition, different sample sizes between the two studies might be a reason for this difference. We have compared our association analyses results of rs2280543 and rs12484776 with the other 3 previous reports (Supplemental Table S4). Among these studies, the directions of effects for both SNPs were basically the same. The only different one was rs2280543 from the study of Bondagji *et al*. This might be due to its small sample size compared to the other 3 studies.

In the UL case only sub-group, we identified significant associations between two targeted SNPs and relevant clinical features of UL. Our data showed that SNP rs2280543 from *BET1L* was significantly associated with the number of fibroid nodes, while the SNP rs12484776 from *TNRC6B* was significantly associated with node size. rs12484776 of *TNRC6B* has been reported to be related to node size (volume) in at least two previous studies based on European populations^14,15^. However, to the best of our knowledge, rs2280543 from *BET1L* has never been reported to be associated with the number of fibroid nodes. Our finding indicated that the TT and CT genotypes of rs2280543 were related to multiple fibroid nodes rather than a single fibroid node in the Han Chinese population. Studies with comparative sample sizes based on other populations are needed to verify these findings in the future.

In this study, we investigated the potential association between UL and two loci, *BET1L* and *TNRC6B*. *BET1L* is a protein coding gene located at 11p15.5. It encodes a protein, BET1L, that facilitates the Golgi vesicular membrane trafficking process^33^. *TNRC6B*, which is located at chromosome 22q13.1, is a tri-nucleotide repeat containing the 6B protein, which was identified to be co-purified with a cytoplasmic HeLa cell protein complex. In addition, the TNRC6B protein was also reported to be required to mediate microRNA-guided mRNA cleavage in HeLa cell culture^34^. Despite these primary studies, no more specific functions of *TNRC6B* have been reported. As a population-based study, it is beyond our scope to investigate the underlying biological mechanisms of these two loci and relate them to the pathogenesis of UL. Experimental studies based on animal models are needed in the future to unravel the roles of both loci in the onset and development of UL.

Both significant SNPs, rs2280543 and rs12484776, seemed to have very limited functional significance based on their RegulomeDB scores, which are derived from regulatory element annotations based on ENCODE data. However, eQTL analyses based on GTEx data showed that both SNPs are significantly associated with the expression of their genes. This eQTL effect was relatively weaker for rs12484776, for which a significant difference in expression was identified in only 1 of 47 human tissues. On the other hand, this effect was more universal and widespread for rs2280543 and its gene, *BET1L*. Expression of *BET1L* was significantly associated with rs2280543 in 15 of 47 human tissues, and the most significant hit in skeletal muscle has a significance level of 10^−18^. Interestingly, a similar eQTL pattern was also reported in the initial GWAS conducted by Cha *et al*.^13^. They also identified that rs2280543 is significantly associated with transcript levels of *BET1L* in three cell types: lymphoblastoid cell lines, peripheral blood mononucleated cells and cortical brains based on *in silico* analysis. The findings of the functional consequence for these candidate SNPs indicate that these SNPs might be more than surrogates but rather have real biological functions contributing to the susceptibility of UL. A potential limitation for our eQTL results is that these data were based on human tissues from normal samples rather than from UL patients. Therefore, we need to be careful in making any premature conclusions. One thing interesting to note is that the protective allele T of rs2280543 from *BET1L* was significantly related to the up-regulated expression of *BET1L* in multiple human tissues. This connection between disease risk of UL and gene expression of *BET1L* might indicate some underlying pathogenesis mechanisms of UL, and further studies are still needed in future to unravel this biological mechanism.

In the study, we have tried our best to restrict population stratification when recruiting subjects by restricting the study subjects with stable living area^35,36^, but the potential population stratification could not be completely ruled out. Moreover, as a candidate gene-based study, we mainly focused on several pre-selected and common tagged polymorphisms. This strategy minimizes the experimental expense at the cost of dropping >90% of the variants of a particular gene. Structural variations and low-frequency and rare variants were not detected in this study. Several recent studies have shown that these undetected DNA variants might play an important role in the susceptibility to complex disorders^37^. Sequencing technology-based studies are needed in the future to systematically evaluate the genetic risk of UL.

In conclusion, in this study, we showed that both *BET1L* and *TNRC6B* contribute to the risk of UL in Chinese women. Significant hits were identified by both single marker-based and haplotype-based analyses. Significant SNPs from *BET1L* and *TNRC6B* were also identified to be significantly associated with the number of fibroid nodes and the size of the nodes, respectively.

## Electronic supplementary material

Supplemental Materials
