# Supplementary material for: Association of BET1L and TNRC6B with uterine leiomyoma risk and its relevant clinical features in Han Chinese population
Source: Sci Rep. 2018 May 9;8:7401. doi: 10.1038/s41598-018-25792-z (PMC5943279; doi:10.1038/s41598-018-25792-z)
Supplement: Supplementary file 1 — Supplemental Materials [file 41598_2018_25792_MOESM1_ESM.docx]

***Title***: Association of *BET1L* and *TNRC6B* with uterine leiomyoma risk and its relevant clinical features in Han Chinese population

***Author names and affiliations***: Bailing Liu ^1,2^, Tao Wang ^3^, Jue Jiang ^1^, Miao Li ^1^, Wenqi Ma ^1^, Haibin Wu ^3^ and Qi Zhou ^1^

^1^ Department of Ultrasound, the Second Affiliated Hospital, Xi'an Jiaotong University, Xi'an, Shaanxi, China;

^2^ Department of Ultrasound, Children's Hospital of Xi'an, Xi'an, Shaanxi, China;

^3^ Department of Pediatrics, Children's Hospital of Xi'an, Xi'an, Shaanxi, China.

***Corresponding Author***:

Qi Zhou, Ph.D. & M.D., Department of Ultrasound, the Second Affiliated Hospital, Xi'an Jiaotong University, 157 Xiwu Road, Xi'an, China, 710004.

Tel: +86-29-87679425, Fax: +86-29-87679323, E-mail: zhouqixjtu@163.com

Supplemental Table S1. General information of our selected SNPs for *BET1L* and *TNRC6B*.

| CHR | POS | SNP | ALLELES | FUNC | GENE | RegDB |
| --- | --- | --- | --- | --- | --- | --- |
| 11 | 175590 | rs59857509 | C/T | unknown | *BET1L* | 5 |
| 11 | 176444 | rs138459941 | C/G | unknown | *BET1L* | 6 |
| 11 | 179664 | rs76859352 | C/T | intron | *BET1L* | 7 |
| 11 | 180225 | rs13377545 | A/G | ncRNA | *BET1L* | 4 |
| 11 | 180231 | rs201265508 | A/G | ncRNA | *BET1L* | 4 |
| 11 | 180258 | rs11502185 | C/T | ncRNA | *BET1L* | 4 |
| 11 | 180261 | rs201966829 | A/T | ncRNA | *BET1L* | 4 |
| 11 | 180318 | rs13377507 | C/T | ncRNA | *BET1L* | 2c |
| 11 | 180483 | rs11502187 | A/T | intron | *BET1L* | 5 |
| 11 | 180689 | rs118152462 | A/G | intron | *BET1L* | 7 |
| 11 | 183529 | rs2686916 | A/C/G | intron | *BET1L* | 7 |
| 11 | 189642 | rs2948211 | C/T | intron,near-gene-5 | *BET1L* | 5 |
| 11 | 191890 | rs58532757 | C/T | intron,near-gene-5 | *BET1L* | 2b |
| 11 | 193863 | rs2294081 | A/G | coding-synon | *BET1L* | 5 |
| 11 | 194331 | rs1108819 | A/C | intron | *BET1L* | 2b |
| 11 | 194639 | rs112126401 | A/G | near-gene-3 | *BET1L* | 4 |
| 11 | 196592 | rs72878023 | A/C | near-gene-5 | *BET1L* | 5 |
| 11 | 196606 | rs28416859 | A/C | near-gene-5 | *BET1L* | 5 |
| 11 | 196912 | rs3825076 | C/G | untranslated-5 | *BET1L* | 5 |
| 11 | 197337 | rs3802984 | A/G | coding-synon | *BET1L* | 4 |
| 11 | 199256 | rs3741411 | A/G | intron | *BET1L* | 1f |
| 11 | 199891 | rs75155656 | A/C | intron | *BET1L* | 2b |
| 11 | 202749 | rs11245992 | A/G | intron | *BET1L* | 4 |
| 11 | 203788 | rs2280543 | C/T | untranslated-3 | *BET1L* | 5 |
| 11 | 204986 | rs4980319 | G/T | untranslated-3 | *BET1L* | 5 |
| 11 | 206089 | rs3782120 | C/T | intron,near-gene-5 | *BET1L* | 2b |
| 11 | 206767 | rs7930823 | C/T | intron,near-gene-5 | *BET1L* | 4 |
| 11 | 207068 | rs117120616 | A/C | intron,near-gene-5 | *BET1L* | 4 |
| 22 | 40065415 | rs5995802 | C/G | intron | *TNRC6B* | 2b |
| 22 | 40089413 | rs58699216 | A/C | intron | *TNRC6B* | 2c |
| 22 | 40095423 | rs10427795 | A/G | intron | *TNRC6B* | 7 |
| 22 | 40102489 | rs9611265 | C/G | intron | *TNRC6B* | 6 |
| 22 | 40114483 | rs74196792 | A/G | intron | *TNRC6B* | 5 |
| 22 | 40115666 | rs1474666 | A/G | intron | *TNRC6B* | 7 |
| 22 | 40121053 | rs12628757 | C/G | intron | *TNRC6B* | 6 |
| 22 | 40133411 | rs6001794 | G/T | intron | *TNRC6B* | 7 |
| 22 | 40142758 | rs117941537 | C/T | intron | *TNRC6B* | 5 |
| 22 | 40147604 | rs11089974 | C/T | intron | *TNRC6B* | 7 |
| 22 | 40151431 | rs117244557 | C/G | intron | *TNRC6B* | 7 |
| 22 | 40215008 | rs28722029 | A/G | intron | *TNRC6B* | 4 |
| 22 | 40215624 | rs138019 | A/G | intron | *TNRC6B* | 6 |
| 22 | 40219272 | rs739182 | G/T | intron | *TNRC6B* | 6 |
| 22 | 40234945 | rs77943556 | A/T | intron | *TNRC6B* | 4 |
| 22 | 40239272 | rs12485003 | A/G | intron | *TNRC6B* | 4 |
| 22 | 40256869 | rs12484776 | A/G | intron | *TNRC6B* | 5 |
| 22 | 40272631 | rs57960171 | A/G | intron | *TNRC6B* | 6 |
| 22 | 40275486 | rs743897 | A/T | intron | *TNRC6B* | 7 |
| 22 | 40306119 | rs201999087 | C/T | intron | *TNRC6B* | 7 |
| 22 | 40310632 | rs2294347 | A/G | intron | *TNRC6B* | 7 |
| 22 | 40312675 | rs2072858 | C/T | intron | *TNRC6B* | 4 |
| 22 | 40319299 | rs139914 | A/G | intron | *TNRC6B* | 5 |
| 22 | 40322096 | rs4821942 | A/G | intron | *TNRC6B* | 5 |
| 22 | 40330179 | rs139919 | C/T | untranslated-3 | *TNRC6B* | 5 |
| 22 | 40330745 | rs139920 | C/T | untranslated-3 | *TNRC6B* | 4 |
| 22 | 40332831 | rs117417000 | C/T | untranslated-3 | *TNRC6B* | 5 |

CHR: chromosome; POS: genomic position; FUNC: function; RegDB: Score of regulomeDB.

Supplemental Table S2. Summarized information for single marker based allelic association tests.

| CHR | SNP | BP | A1 | F_A | F_U | HWE_U | χ^2^ | *P* | OR |
| --- | --- | --- | --- | --- | --- | --- | --- | --- | --- |
| 11 | rs59857509 | 175590 | T | 0.32 | 0.33 | 1.00 | 0.34 | 0.5587 | 0.96 |
| 11 | rs138459941 | 176444 | G | 0.22 | 0.21 | 1.00 | 0.21 | 0.6444 | 1.04 |
| 11 | rs76859352 | 179664 | T | 0.42 | 0.41 | 0.37 | 0.21 | 0.6483 | 1.03 |
| 11 | rs13377545 | 180225 | A | 0.23 | 0.24 | 0.77 | 0.26 | 0.6116 | 0.96 |
| 11 | rs201265508 | 180231 | G | 0.10 | 0.09 | 1.00 | 0.48 | 0.4882 | 1.08 |
| 11 | rs11502185 | 180258 | C | 0.12 | 0.13 | 0.71 | 0.49 | 0.4838 | 0.93 |
| 11 | rs201966829 | 180261 | T | 0.12 | 0.13 | 1.00 | 0.24 | 0.6242 | 0.95 |
| 11 | rs13377507 | 180318 | T | 0.34 | 0.33 | 0.72 | 0.18 | 0.6700 | 1.03 |
| 11 | rs11502187 | 180483 | A | 0.37 | 0.38 | 0.65 | 0.14 | 0.7100 | 0.97 |
| 11 | rs118152462 | 180689 | A | 0.08 | 0.08 | 0.59 | 0.52 | 0.4727 | 0.92 |
| 11 | rs2686916 | 183529 | C | 0.42 | 0.41 | 0.91 | 0.21 | 0.6483 | 1.03 |
| 11 | rs2948211 | 189642 | T | 0.16 | 0.17 | 0.77 | 0.68 | 0.4113 | 0.93 |
| 11 | rs58532757 | 191890 | C | 0.41 | 0.42 | 0.74 | 0.19 | 0.6612 | 0.97 |
| 11 | rs2294081 | 193863 | T | 0.17 | 0.16 | 0.77 | 0.32 | 0.5738 | 1.05 |
| 11 | rs1108819 | 194331 | A | 0.10 | 0.10 | 0.29 | 0.13 | 0.7227 | 0.96 |
| 11 | rs112126401 | 194639 | A | 0.07 | 0.07 | 0.84 | 0.43 | 0.5136 | 0.92 |
| 11 | rs72878023 | 196592 | C | 0.15 | 0.14 | 0.50 | 0.70 | 0.4027 | 1.08 |
| 11 | rs28416859 | 196606 | C | 0.20 | 0.21 | 0.51 | 0.10 | 0.7523 | 0.97 |
| 11 | rs3825076 | 196912 | G | 0.12 | 0.13 | 0.90 | 0.34 | 0.5572 | 0.94 |
| 11 | rs3802984 | 197337 | G | 0.33 | 0.32 | 0.81 | 0.23 | 0.6293 | 1.04 |
| 11 | rs3741411 | 199256 | G | 0.45 | 0.44 | 0.91 | 0.45 | 0.5034 | 1.05 |
| 11 | rs75155656 | 199891 | A | 0.41 | 0.40 | 0.74 | 0.19 | 0.6607 | 1.03 |
| 11 | rs11245992 | 202749 | A | 0.12 | 0.13 | 0.81 | 0.30 | 0.5811 | 0.95 |
| 11 | rs2280543 | 203788 | T | 0.10 | 0.15 | 0.33 | 18.32 | 1.87E-05 | 0.64 |
| 11 | rs4980319 | 204986 | C | 0.15 | 0.16 | 1.00 | 0.36 | 0.5498 | 0.95 |
| 11 | rs3782120 | 206089 | A | 0.16 | 0.15 | 0.91 | 0.65 | 0.4218 | 1.08 |
| 11 | rs7930823 | 206767 | A | 0.28 | 0.29 | 0.60 | 0.18 | 0.6749 | 0.97 |
| 11 | rs117120616 | 207068 | A | 0.07 | 0.07 | 0.83 | 0.14 | 0.7086 | 0.95 |
| 22 | rs5995802 | 40065415 | G | 0.42 | 0.43 | 0.51 | 0.05 | 0.8222 | 0.99 |
| 22 | rs58699216 | 40089413 | C | 0.08 | 0.07 | 0.69 | 0.34 | 0.5576 | 1.08 |
| 22 | rs10427795 | 40095423 | A | 0.09 | 0.09 | 1.00 | 0.25 | 0.6166 | 1.06 |
| 22 | rs9611265 | 40102489 | C | 0.24 | 0.23 | 0.94 | 0.29 | 0.5934 | 1.04 |
| 22 | rs74196792 | 40114483 | A | 0.10 | 0.10 | 0.65 | 0.10 | 0.7501 | 0.96 |
| 22 | rs1474666 | 40115666 | A | 0.19 | 0.19 | 0.86 | 0.13 | 0.7235 | 1.03 |
| 22 | rs12628757 | 40121053 | G | 0.09 | 0.09 | 1.00 | 0.18 | 0.6731 | 1.05 |
| 22 | rs6001794 | 40133411 | T | 0.25 | 0.24 | 0.94 | 0.24 | 0.6224 | 1.04 |
| 22 | rs117941537 | 40142758 | C | 0.07 | 0.07 | 0.43 | 0.33 | 0.5650 | 0.93 |
| 22 | rs11089974 | 40147604 | T | 0.25 | 0.24 | 0.94 | 0.32 | 0.5706 | 1.05 |
| 22 | rs117244557 | 40151431 | G | 0.06 | 0.07 | 0.66 | 0.07 | 0.7956 | 0.97 |
| 22 | rs28722029 | 40215008 | A | 0.24 | 0.25 | 0.83 | 0.09 | 0.7650 | 0.98 |
| 22 | rs138019 | 40215624 | A | 0.10 | 0.10 | 0.38 | 0.55 | 0.4564 | 0.92 |
| 22 | rs739182 | 40219272 | T | 0.34 | 0.38 | 0.31 | 7.92 | 0.0049 | 0.82 |
| 22 | rs77943556 | 40234945 | T | 0.11 | 0.12 | 1.00 | 0.40 | 0.5275 | 0.94 |
| 22 | rs12485003 | 40239272 | A | 0.11 | 0.10 | 0.88 | 0.37 | 0.5408 | 1.07 |
| 22 | rs12484776 | 40256869 | G | 0.29 | 0.22 | 0.53 | 19.73 | 8.91E-06 | 1.40 |
| 22 | rs57960171 | 40272631 | A | 0.11 | 0.12 | 0.70 | 0.19 | 0.6668 | 0.96 |
| 22 | rs743897 | 40275486 | T | 0.08 | 0.08 | 0.86 | 0.26 | 0.6104 | 0.94 |
| 22 | rs201999087 | 40306119 | T | 0.07 | 0.07 | 0.83 | 0.21 | 0.6433 | 0.94 |
| 22 | rs2294347 | 40310632 | A | 0.08 | 0.07 | 0.84 | 0.35 | 0.5563 | 1.08 |
| 22 | rs2072858 | 40312675 | C | 0.40 | 0.35 | 1.00 | 7.63 | 0.0057 | 1.21 |
| 22 | rs139914 | 40319299 | G | 0.30 | 0.29 | 0.44 | 0.09 | 0.7619 | 1.02 |
| 22 | rs4821942 | 40322096 | A | 0.20 | 0.21 | 0.93 | 0.08 | 0.7754 | 0.98 |
| 22 | rs139919 | 40330179 | C | 0.24 | 0.24 | 0.46 | 0.07 | 0.7885 | 0.98 |
| 22 | rs139920 | 40330745 | T | 0.31 | 0.32 | 0.76 | 0.24 | 0.6269 | 0.97 |
| 22 | rs117417000 | 40332831 | T | 0.08 | 0.07 | 0.84 | 0.30 | 0.5862 | 1.07 |

F_A: allele frequency of patients; F_U: allele frequency of controls.

Supplemental Table S3. eQTL results for rs2280543 and rs12484776 from 47 human tissues.

| GENE | SNP | *P* | Effect Size | T-Statistic | Standard Error | Tissue |
| --- | --- | --- | --- | --- | --- | --- |
| *BET1L* | rs2280543 | 1.70E-18 | 0.73 | 9.2 | 0.079 | Muscle - Skeletal |
| *BET1L* | rs2280543 | 1.10E-11 | 0.53 | 7.1 | 0.075 | Artery - Tibial |
| *BET1L* | rs2280543 | 5.10E-11 | 0.47 | 6.8 | 0.07 | Skin - Sun Exposed (Lower leg) |
| *BET1L* | rs2280543 | 5.40E-10 | 0.58 | 6.4 | 0.09 | Esophagus - Mucosa |
| *BET1L* | rs2280543 | 1.20E-09 | 0.5 | 6.3 | 0.079 | Esophagus - Muscularis |
| *BET1L* | rs2280543 | 5.20E-08 | 0.58 | 5.6 | 0.1 | Artery - Aorta |
| *BET1L* | rs2280543 | 1.40E-07 | 0.45 | 5.4 | 0.083 | Adipose - Subcutaneous |
| *BET1L* | rs2280543 | 1.10E-06 | 0.43 | 5 | 0.085 | Nerve - Tibial |
| *BET1L* | rs2280543 | 1.40E-06 | 0.57 | 5 | 0.12 | Breast - Mammary Tissue |
| *BET1L* | rs2280543 | 9.50E-06 | 0.91 | 4.8 | 0.19 | Brain - Spinal cord (cervical c-1) |
| *BET1L* | rs2280543 | 1.60E-05 | 0.38 | 4.4 | 0.086 | Skin - Not Sun Exposed (Suprapubic) |
| *BET1L* | rs2280543 | 1.20E-04 | 0.33 | 3.9 | 0.085 | Thyroid |
| *BET1L* | rs2280543 | 2.80E-04 | 0.43 | 3.7 | 0.12 | Esophagus - Gastroesophageal Junction |
| *BET1L* | rs2280543 | 4.00E-04 | 0.46 | 3.6 | 0.13 | Colon - Sigmoid |
| *TNRC6B* | rs12484776 | 4.60E-04 | -0.17 | -3.5 | 0.048 | Esophagus - Muscularis |
| *BET1L* | rs2280543 | 6.80E-04 | 0.57 | 3.5 | 0.16 | Adrenal Gland |
| *BET1L* | rs2280543 | 0.0016 | 0.64 | 3.3 | 0.2 | Ovary |
| *BET1L* | rs2280543 | 0.0019 | -0.57 | -3.2 | 0.18 | Brain - Nucleus accumbens (basal ganglia) |
| *BET1L* | rs2280543 | 0.0028 | -0.34 | -3 | 0.11 | Pituitary |
| *TNRC6B* | rs12484776 | 0.0030 | 0.24 | 3 | 0.079 | Pancreas |
| *BET1L* | rs2280543 | 0.0068 | 0.36 | 2.7 | 0.13 | Testis |
| *TNRC6B* | rs12484776 | 0.0074 | 0.14 | 2.7 | 0.05 | Artery - Aorta |
| *BET1L* | rs2280543 | 0.0078 | 0.57 | 2.7 | 0.21 | Uterus |
| *BET1L* | rs2280543 | 0.0089 | 0.28 | 2.6 | 0.11 | Whole Blood |
| *BET1L* | rs2280543 | 0.0100 | -0.52 | -2.6 | 0.2 | Brain - Frontal Cortex (BA9) |
| *BET1L* | rs2280543 | 0.0150 | 0.38 | 2.5 | 0.15 | Artery - Coronary |
| *TNRC6B* | rs12484776 | 0.0180 | 0.089 | 2.4 | 0.038 | Adipose - Visceral (Omentum) |
| *TNRC6B* | rs12484776 | 0.0240 | 0.14 | 2.3 | 0.062 | Brain - Cerebellum |
| *BET1L* | rs2280543 | 0.0300 | 0.42 | 2.2 | 0.19 | Small Intestine - Terminal Ileum |
| *BET1L* | rs2280543 | 0.0330 | 0.21 | 2.1 | 0.096 | Stomach |
| *BET1L* | rs2280543 | 0.0350 | 0.25 | 2.1 | 0.12 | Adipose - Visceral (Omentum) |
| *BET1L* | rs2280543 | 0.0360 | 0.38 | 2.1 | 0.18 | Prostate |
| *TNRC6B* | rs12484776 | 0.0380 | 0.066 | 2.1 | 0.032 | Artery - Tibial |
| *TNRC6B* | rs12484776 | 0.0450 | 0.13 | 2 | 0.063 | Artery - Coronary |
| *BET1L* | rs2280543 | 0.0630 | 0.2 | 1.9 | 0.11 | Heart - Left Ventricle |
| *TNRC6B* | rs12484776 | 0.0630 | 0.28 | 1.9 | 0.15 | Minor Salivary Gland |
| *TNRC6B* | rs12484776 | 0.0680 | 0.15 | 1.8 | 0.079 | Brain - Cerebellar Hemisphere |
| *BET1L* | rs2280543 | 0.0880 | -0.41 | -1.7 | 0.24 | Brain - Cerebellum |
| *TNRC6B* | rs12484776 | 0.0910 | -0.2 | -1.7 | 0.11 | Ovary |
| *BET1L* | rs2280543 | 0.0950 | 0.15 | 1.7 | 0.091 | Lung |
| *TNRC6B* | rs12484776 | 0.0980 | 0.12 | 1.7 | 0.073 | Spleen |
| *TNRC6B* | rs12484776 | 0.1300 | 0.049 | 1.5 | 0.033 | Thyroid |
| *TNRC6B* | rs12484776 | 0.1700 | -0.056 | -1.4 | 0.041 | Muscle - Skeletal |
| *TNRC6B* | rs12484776 | 0.1800 | 0.037 | 1.3 | 0.028 | Lung |
| *TNRC6B* | rs12484776 | 0.2700 | 0.11 | 1.1 | 0.099 | Prostate |
| *TNRC6B* | rs12484776 | 0.2700 | 0.036 | 1.1 | 0.032 | Skin - Sun Exposed (Lower leg) |
| *TNRC6B* | rs12484776 | 0.3000 | -0.093 | -1 | 0.09 | Brain - Anterior cingulate cortex (BA24) |
| *TNRC6B* | rs12484776 | 0.3300 | -0.091 | -0.98 | 0.093 | Brain - Hippocampus |
| *TNRC6B* | rs12484776 | 0.3300 | -0.088 | -0.99 | 0.09 | Brain - Hypothalamus |
| *BET1L* | rs2280543 | 0.3500 | 0.16 | 0.94 | 0.17 | Liver |
| *TNRC6B* | rs12484776 | 0.3500 | 0.084 | 0.94 | 0.089 | Adrenal Gland |
| *TNRC6B* | rs12484776 | 0.3700 | -0.07 | -0.9 | 0.078 | Brain - Caudate (basal ganglia) |
| *TNRC6B* | rs12484776 | 0.3700 | -0.082 | -0.9 | 0.09 | Brain - Frontal Cortex (BA9) |
| *TNRC6B* | rs12484776 | 0.3700 | -0.032 | -0.9 | 0.036 | Nerve - Tibial |
| *TNRC6B* | rs12484776 | 0.3800 | -0.063 | -0.89 | 0.071 | Brain - Cortex |
| *TNRC6B* | rs12484776 | 0.3800 | -0.059 | -0.87 | 0.068 | Esophagus - Gastroesophageal Junction |
| *BET1L* | rs2280543 | 0.4000 | 0.18 | 0.84 | 0.21 | Brain - Anterior cingulate cortex (BA24) |
| *BET1L* | rs2280543 | 0.4200 | 0.064 | 0.8 | 0.08 | Pancreas |
| *TNRC6B* | rs12484776 | 0.4200 | -0.067 | -0.81 | 0.083 | Cells - EBV-transformed lymphocytes |
| *BET1L* | rs2280543 | 0.4500 | -0.25 | -0.76 | 0.32 | Brain - Cortex |
| *BET1L* | rs2280543 | 0.4600 | -0.14 | -0.74 | 0.19 | Brain - Hippocampus |
| *TNRC6B* | rs12484776 | 0.4700 | -0.049 | -0.72 | 0.068 | Pituitary |
| *TNRC6B* | rs12484776 | 0.4900 | -0.059 | -0.69 | 0.085 | Colon - Sigmoid |
| *TNRC6B* | rs12484776 | 0.4900 | -0.02 | -0.69 | 0.029 | Whole Blood |
| *TNRC6B* | rs12484776 | 0.5300 | 0.045 | 0.63 | 0.071 | Liver |
| *BET1L* | rs2280543 | 0.5400 | 0.13 | 0.61 | 0.21 | Vagina |
| *TNRC6B* | rs12484776 | 0.5400 | 0.026 | 0.61 | 0.042 | Esophagus - Mucosa |
| *TNRC6B* | rs12484776 | 0.5500 | -0.035 | -0.6 | 0.058 | Testis |
| *TNRC6B* | rs12484776 | 0.5600 | 0.06 | 0.58 | 0.1 | Brain - Substantia nigra |
| *TNRC6B* | rs12484776 | 0.6000 | -0.053 | -0.52 | 0.1 | Brain - Putamen (basal ganglia) |
| *TNRC6B* | rs12484776 | 0.6000 | 0.051 | 0.53 | 0.097 | Vagina |
| *TNRC6B* | rs12484776 | 0.6400 | 0.054 | 0.47 | 0.11 | Brain - Amygdala |
| *BET1L* | rs2280543 | 0.6500 | 0.065 | 0.45 | 0.14 | Heart - Atrial Appendage |
| *BET1L* | rs2280543 | 0.6600 | 0.099 | 0.45 | 0.22 | Brain - Substantia nigra |
| *BET1L* | rs2280543 | 0.6700 | 0.08 | 0.42 | 0.19 | Brain - Amygdala |
| *TNRC6B* | rs12484776 | 0.6900 | -0.049 | -0.4 | 0.12 | Uterus |
| *TNRC6B* | rs12484776 | 0.7500 | -0.025 | -0.33 | 0.076 | Brain - Nucleus accumbens (basal ganglia) |
| *TNRC6B* | rs12484776 | 0.7800 | 0.017 | 0.28 | 0.061 | Heart - Atrial Appendage |
| *TNRC6B* | rs12484776 | 0.7800 | -0.021 | -0.28 | 0.075 | Small Intestine - Terminal Ileum |
| *BET1L* | rs2280543 | 0.8100 | -0.048 | -0.25 | 0.19 | Cells - EBV-transformed lymphocytes |
| *BET1L* | rs2280543 | 0.8200 | 0.046 | 0.23 | 0.2 | Brain - Putamen (basal ganglia) |
| *TNRC6B* | rs12484776 | 0.8600 | 0.008 | 0.18 | 0.044 | Heart - Left Ventricle |
| *BET1L* | rs2280543 | 0.8700 | 0.029 | 0.16 | 0.17 | Brain - Cerebellar Hemisphere |
| *BET1L* | rs2280543 | 0.8800 | 0.022 | 0.15 | 0.15 | Brain - Caudate (basal ganglia) |
| *TNRC6B* | rs12484776 | 0.8800 | 0.006 | 0.15 | 0.04 | Adipose - Subcutaneous |
| *BET1L* | rs2280543 | 0.9100 | 0.022 | 0.11 | 0.2 | Brain - Hypothalamus |
| *TNRC6B* | rs12484776 | 0.9100 | -0.0057 | -0.12 | 0.048 | Cells - Transformed fibroblasts |
| *BET1L* | rs2280543 | 0.9200 | -0.018 | -0.095 | 0.19 | Spleen |
| *TNRC6B* | rs12484776 | 0.9200 | 0.0045 | 0.11 | 0.042 | Skin - Not Sun Exposed (Suprapubic) |
| *TNRC6B* | rs12484776 | 0.9200 | 0.0069 | 0.098 | 0.071 | Stomach |
| *BET1L* | rs2280543 | 0.9400 | -0.0081 | -0.073 | 0.11 | Cells - Transformed fibroblasts |
| *TNRC6B* | rs12484776 | 0.9700 | -0.0041 | -0.039 | 0.1 | Brain - Spinal cord (cervical c-1) |
| *TNRC6B* | rs12484776 | 0.9900 | -0.00064 | -0.014 | 0.047 | Breast - Mammary Tissue |

Supplemental Table S4. Results of association analyses for rs2280543 and rs12484776 with UL and its related phenotype.

| SNP | Gene | Risk Allele | OR | *P-*values | Sample Size | References |
| --- | --- | --- | --- | --- | --- | --- |
| rs2280543 | *BET1L* | T | 0.64 | 1.87×10^-5^ | 2,055 | Our study |
|  |  | C | 1.39 | 3.82 × 10^−12^ | 3,035 | Cha *et al.* |
|  |  | T | 0.50 | 0.023 | 2,005 | Edwards *et al.* |
|  |  | T | 1.52 | 0.31 | 217 | Bondagji *et al.* |
| rs12484776 | *TNRC6B* | G | 1.40 | 8.91×10^-6^ | 2,055 | Our study |
|  |  | G | 1.23 | 2.79 × 10^−12^ | 3,035 | Cha *et al.* |
|  |  | G | 2.19 | 0.031 | 2,005 | Edwards *et al.* |
|  |  | G | 5.48 | 0.01 | 217 | Bondagji *et al.* |


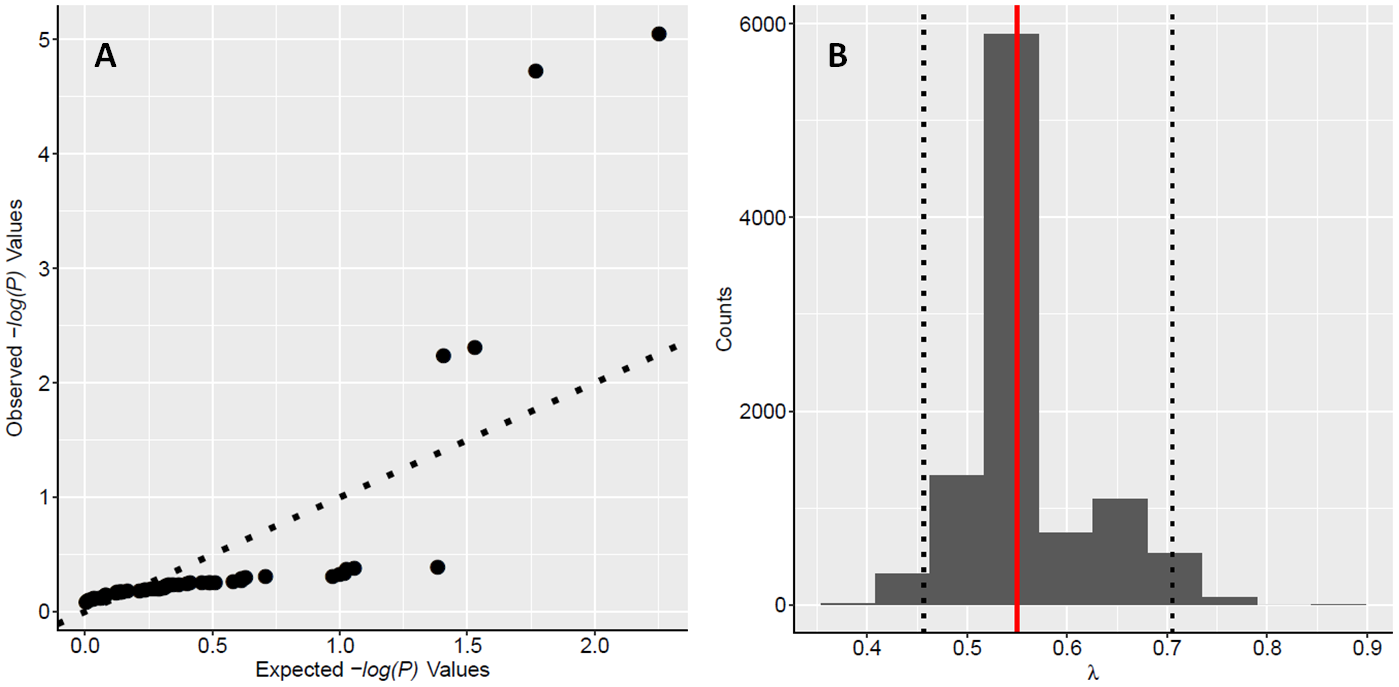


Supplemental Figure S1. Genomic controls applied to estimating the potential population stratifications. A. Q-Q plot made from the results of single marker based association analyses. B. Histogram of inflation factor λ obtained through 10,000 bootstrapping. Red line and dotted lines were observedλ and its 95% confidence interval, respectively.
